# Improving Compliance With Valid Oxygen Prescriptions for Surgical Inpatients in a District General Hospital: A Single-Centre Quality Improvement Study

**DOI:** 10.7759/cureus.71600

**Published:** 2024-10-16

**Authors:** Sulaiman Hussain, Zina Mobarak, Shaher Yar Ahmad, Haris Shoaib, Anse Arif, Mooyad A Ahmed

**Affiliations:** 1 Department of Surgery, Royal Blackburn Teaching Hospital, Blackburn, GBR; 2 Department of Surgery, Manchester Royal Infirmary, Manchester, GBR; 3 Department of Trauma and Orthopaedics, Royal Preston Hospital, Preston, GBR

**Keywords:** general surgery complication, oxygen prescription, peri-operative medicine, post-op complications, prescription audit

## Abstract

Introduction

Medical oxygen is a drug and, as such, must be correctly prescribed according to British Thoracic Society (BTS) guidelines. These guidelines state that a valid prescription must include a target oxygen saturation range, and that all inpatients should have a valid oxygen prescription. A 2008 BTS audit revealed only 32% of patients receiving oxygen had valid prescriptions, and a 2015 re-audit showed improvement to 57.5%, still below the national 95% target. Unregulated oxygen administration can lead to complications such as hypoxia, hyperoxia, and increased healthcare costs. Our quality improvement project (QIP) aimed to improve adherence to BTS guidelines on two general surgical wards at Royal Blackburn Teaching Hospital, aiming for complete adherence.

Methods

A quality improvement study was conducted using electronic patient records (EPRs). In the baseline audit, data was collected on patients over a one-week period. Thirty-three patients were included, with information on age, oxygen prescriptions, and Chronic Obstructive Pulmonary Disease (COPD) status recorded. Four interventions were then implemented: the addition of reminders to handover sheets, visual prompts around the ward, announcements during nursing huddles, and WhatsApp reminders to the ward doctors. A re-audit was conducted after the interventions, including 31 patients, and data was compared using the Chi-squared test.

Results

In the baseline audit, 18% of patients had oxygen prescribed. Following the interventions, this rose to 54.8% (χ²(1, N=64) = 9.3, p < 0.01), and as such, was statistically significant. Among patients requiring oxygen, compliance improved from 0% to 90.9%.

Discussion

The interventions significantly improved oxygen prescription compliance, demonstrating the effectiveness of simple, targeted measures. The inclusion of the multidisciplinary team (MDT) was crucial, as both nurses and doctors play essential roles in oxygen delivery. However, compliance remained below the BTS target of 100%. Limitations include not assessing the impact of individual interventions and analyzing only two points in time. Future audits should focus on targeting prescribers early in admissions and integrate electronic systems for automated prescription prompts. Spot audits could help ensure long-term success.

Conclusion

This QIP improved compliance with BTS oxygen prescribing guidelines at Royal Blackburn Teaching Hospital, from 18% to 54.8%. Engaging the MDT and using reminders increased compliance, but further efforts are needed to achieve the 100% target. Future interventions should focus on EPR integration, ongoing education, and further audit cycles for sustained improvement.

## Introduction

Medical oxygen is a recognised drug and as such should be prescribed correctly by a clinician [1]. The British Thoracic Society (BTS) regularly publishes audited and updated guidelines pertaining to the use of oxygen in health and emergency care for adults over the age of 16, including its use in the peri-operative setting. The BTS clarifies that an oxygen prescription needs to have a target saturation range to be deemed valid [2]. In 2008, the BTS performed a national audit that found only 32% of patients receiving therapeutic oxygen had it prescribed correctly [3]. The BTS set a national improvement objective, aiming for 95% of patients using oxygen to have a valid prescription with a target saturation range. A 2015 re-audit demonstrated an improvement to 57.5%, still far from meeting their target [4]. Such non-compliance would be unacceptable for any other drug. As such, the current BTS guidelines recommend that all inpatients should have oxygen prescribed on the drug chart or electronic prescribing system, with a target saturation range, and monitoring via pulse oximetry in surgical inpatients.

Our quality improvement project (QIP) focuses on meeting the standard of valid oxygen prescriptions set by the BTS on two general surgical wards at Royal Blackburn Teaching Hospital, East Lancashire NHS Trust. Previously, our trust has conducted similar QIPs; however, adherence to the standards set has been poor.

Oxygen is used to treat hypoxemia and tissue hypoxia, though it can be used for other pathologies without an oxygen deficit [5]. Profound hypoxaemia can lead to organ failure and death. Equally, hyperoxia can lead to atelectasis, increased reactive oxygen species, and worsen ischemic heart disease in the peri-operative setting, contributing to increased medium/long-term mortality for these patients [2,6]. Studies have shown that early intervention to increase oxygen delivery to tissues in critically ill patients, including high-risk surgical patients, reduces organ failure, reduces the length of ICU stays, and improves survival; importantly, these studies did not reveal any benefit from supra-physiological oxygen delivery [7-10]. Furthermore, oxygen wastage in hyperoxia and prolonged hospital stays owing to hypoxia postoperatively increase the financial burden on hospital trusts [11]. Thus, it is important to optimise oxygen delivery with valid oxygen prescriptions.

Target saturations in the peri-operative setting for most surgical patients are 94-98% to prevent hypoxia and hyperoxia respectively. However, those at risk of hypercapnic respiratory failure have target saturations set at 88-92%. Unregulated oxygen administration can worsen the existing ventilation-perfusion mismatch and alveolar hypoventilation in patients with hypercapnic respiratory failure through a variety of complex mechanisms, ultimately leading to worsening hypoxia [2]. A study by Decalmer S and O'Driscoll BR revealed that hypercapnia is a more common finding than hypoxia on surgical wards, highlighting the importance of a valid oxygen prescription [12].

## Materials and methods

Study design

This was a quality improvement study where data were collected via electronic patient records. Two audit cycles were completed, with the re-audit being completed following the implementation of targeted interventions. The audit was registered with the local audit and clinical governance team at Royal Blackburn Teaching Hospital.

Baseline audit

For the baseline audit, data on adherence to BTS oxygen prescribing guidelines were collected on patients over a one-week period (February 5-9, 2024), on two general surgical wards at Royal Blackburn Teaching Hospital. General surgery was chosen as the specialty of interest as it comprises the largest number of inpatients among the surgical specialties at Royal Blackburn Teaching Hospital. On these two general surgical wards, all patients over 18 admitted under general surgery were included. Patients under the age of 18, and patients admitted as medical outliers, were excluded. Their medical records were analyzed, with data collected including: age, validity of the oxygen prescription on the drug chart, oxygen administration during admission, chronic obstructive pulmonary disease (COPD) status, and primary diagnosis. A valid oxygen prescription was defined as one that stated a target oxygen saturation range and an acceptable oxygen prescription on the electronic patient record. Data were anonymized, tabulated, and analyzed. Thirty-three patients were included in the baseline audit.

Interventions/plan-do-study-act (PDSA) cycles

Following the initial data collection, the PDSA framework was employed to identify areas for improvement and design interventions to improve compliance with BTS guidelines. Four interventions were devised and carried out.

The first intervention was the addition of a heading text reminder to clinical handover sheets. The handover sheet is updated each day for each of the two wards by the ward doctors. This handover sheet includes a list of all the patients on each surgical ward, with relevant clinical information and associated outstanding tasks. A text reminder was added to the top of the template for this document, so each day when it was viewed by the doctors, it would act as a reminder to prescribe oxygen for each new admission to the ward.

**Figure 1:**
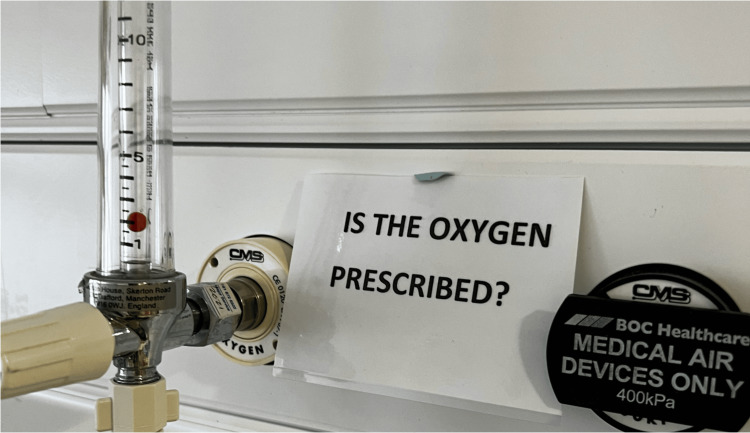
Reminder poster on oxygen gauge at a patient's bed.

Visual prompts were put up around the ward, next to each oxygen gauge on the ward (Figure 1). This was designed to be simple and eye-catching. For the nursing team, this would remind them to tell doctors if they noticed oxygen was not prescribed on the drug chart so that it could be prescribed accordingly, an even more pertinent reminder if the oxygen was actively being used.

The nursing staff on each ward have a team meeting or ‘huddle’ every morning where each patient is briefly discussed. An announcement was made every day for a week during this huddle to alert nursing staff about the importance of oxygen prescription, to inform doctors to prescribe oxygen, and to let nurses know to tell doctors if they notice that oxygen has not been prescribed. The announcement also allowed us to draw attention to the visual prompts that had been put up in the ward. This intervention was chosen due to its success in previous oxygen prescribing QIPs [13,14].

Finally, announcements were made to FY1 doctors who work in the general surgery department via WhatsApp group chats, as this was shown to be an effective intervention in a previous QIP due to it specifically targeting those who have the ability to prescribe oxygen [14]. The message included information on the aims of the QIP, the importance of prescribing oxygen, and a reminder to ensure oxygen is prescribed.

Re-audit

To evaluate the impact of the implementations, data were collected over a similar one-week period (March 25-29, 2024) on patients on the same two general surgical wards. The same methodology as the baseline audit was followed, with the same data variables being used to allow for direct comparison. Thirty-one patients were included in the re-audit. The pre-intervention and post-intervention data were then analyzed and compared using the Chi-squared test to evaluate the change.

## Results

Thirty-three patients were included in the initial audit. The median age was 71 (IQR: 57.5, 82.5), with no patients having a diagnosis of COPD. The most common diagnosis was post-operation (14/33, 42.4%), followed by infection (8/33, 24.2%), malignancy (7/33, 21.2%), and other (4/33, 12.1%). Six patients (18%) had oxygen prescribed on their drug chart, with none requiring oxygen during admission (Tables 1-2).

**Table 1:** Characteristics of patients included in both audits. n: Sample size; COPD: Chronic obstructive pulmonary disease.

Patient characteristics	Initial Audit (n=33)	Re-Audit (n=31)
Median age	71 (IQR 57.5, 82.5)	63 (IQR 54, 72)
COPD diagnosis	0	3
Post-operative	14	15
Infection	8	4
Malignancy	7	10
Other diagnoses	4	2

**Table 2:** Outcome data from both audits. EPR: Electronic patient record; n: Sample size; N/A: Not applicable.

	Initial Audit (n=33)	Re-Audit (n=31)
Oxygen prescribed on EPR	6	17
Required oxygen during admission	0	11
Oxygen prescribed if required oxygen	N/A	10

In the re-audit, 31 patients were included, with the median age being 63 (IQR 54, 72). Three patients (9.7%) had a diagnosis of COPD. The most common diagnosis was post-operation (15/31, 48.4%), followed by malignancy (10/31, 32.3%), infection (4/31, 12.9%), and other (2/31, 6.5%). Seventeen patients (54.8%) had oxygen prescribed on their drug chart. Eleven patients (35.5%) required oxygen, of whom 10 (90.9%) had oxygen prescribed.

There was a significant increase from 18% of patients having oxygen prescribed to 54.8% post-intervention. This increase was statistically significant, χ²(1, N = 64) = 9.3, p < 0.01.

## Discussion

The re-audit data showed that overall, our interventions were effective at increasing compliance with BTS oxygen prescribing guidelines, as oxygen prescriptions rose from 18% to 54.8%. This demonstrates that simple interventions can have a positive impact, which is further supported by previous oxygen prescription QIPs [13]. However, while compliance increased, there is still room for further improvement, with just under half of the patients not having oxygen prescribed in the re-audit. Valuable lessons can be taken from this to inform future iterations of this project and to ensure even better compliance rates.

One strength of this audit is that different parts of the healthcare team were included in the interventions. When designing interventions, it was crucial to think about the pathway from prescribing oxygen to administering it. Previous audits have targeted only doctors because they are the primary oxygen prescribers; however, including other members of the multidisciplinary team (MDT) is essential as they are also involved in the delivery of oxygen to patients. This has proved to be successful in our audit, with having the wider MDT aware of the audit being beneficial to the outcome. Our interventions targeted both doctors who worked regularly on the two wards and doctors involved in admitting patients, meaning different settings of prescribing oxygen were addressed. The identity of the prescribers and the time of prescription (whether at the time of admission or later) was not collected, however, in order to assess where the interventions were most effective. As all patients admitted under general surgery in an emergency setting come through Accident and Emergency (A&E) followed by the emergency surgical unit before being admitted on the general surgical wards, it would have been useful to have put an emphasis on implementing interventions for A&E clinicians, in order to target patients as soon as they enter the hospital, and, as a result, improve compliance in all general surgical patients across all wards.

One limitation of this study is that a re-audit was not completed after each intervention, meaning the impact of each intervention was not assessed individually. This raises the issue that it is unknown exactly which interventions made an impact and which did not, meaning if another hospital trust wished to improve their oxygen prescription compliance based on our project, it would not be known which interventions should be used. In the future, multiple audit cycles could be carried out, with each one involving one intervention at a time, to allow for better analysis and comparisons between interventions.

Furthermore, data were extracted at only two points in time - before and after the interventions. This means that it is unknown whether there has been a sustained change in oxygen prescribing on the wards, as it is possible that the doctors on the wards were more aware of the interventions when they had just been put in place, but forgot about them as time went on. Most doctors working on these general surgical wards rotate every 4-6 months, meaning that many of the doctors on the ward at the time of the audit no longer work there. Including other members of the MDT as we did, such as nurses, may have helped to ensure a sustained change, as they are more likely to be permanent staff on the ward. However, in order to truly assess if compliance is still improved in comparison to the pre-audit, more data collection is required, and spot audits could be conducted at different points in time.

For the future, interventions could incorporate the EPR system into the interventions. Our hospital trust recently implemented an EPR system where all prescribing has been made electronic; however, our implementations did not include this. Discussion with the hospital trust’s IT department could result in a prompt or reminder, alerting doctors to prescribe oxygen when admitting patients, which would ensure all patients admitted to the hospital have this on their drug chart. This prompt has already been shown to be effective for venous thromboembolism prophylaxis prescription and could result in a sustained change if done for oxygen prescription.

## Conclusions

From the literature, it is evident that adherence to oxygen prescription guidelines in secondary healthcare has been a longstanding issue. This audit is a step in addressing this problem, successfully demonstrating the effectiveness of a team of junior doctors in improving oxygen prescription compliance at Royal Blackburn Teaching Hospital. The utilization of the MDT played a key role in ensuring that oxygen was prescribed correctly, while visual prompts and reminders proved to be effective tools. To yield a trust-wide positive outcome, a larger audit would need to take place, with these interventions being implemented beyond the two surgical wards involved in this study.

Despite the success, there is still significant room for improvement to further increase oxygen prescription compliance, with several limitations being identified. The BTS guidelines require 100% compliance with valid oxygen prescriptions for all inpatients, a statistic that was not achieved in this audit. Future cycles of this audit should ensure data collection after each intervention, target prescribers early in the patient admission process, and utilize the electronic patient record system. Moving forward, we will continue to collaborate with the trust to implement these strategies to drive sustained improvements in oxygen prescribing practices across the hospital.
